# Characterization and Enzymatic Potential of Bacteria and Fungi From Mwakirunge Dumpsite, Kenya

**DOI:** 10.1155/ijm/7818433

**Published:** 2025-04-21

**Authors:** Beryle Atieno Okoth, Huxley Mae Makonde, Carren Moraa Bosire, Cromwell Mwiti Kibiti

**Affiliations:** Department of Pure and Applied Sciences, Technical University of Mombasa, Mombasa, Kenya

**Keywords:** bacteria, biodegradation, extracellular enzymes, fungi, solid wastes

## Abstract

Accumulation of solid waste is a major global challenge. The conventional waste disposal methods are often ineffective in mitigating solid waste pollution, highlighting the need for other sustainable alternatives. This study is aimed at isolating and identifying potential waste-degrading microorganisms from Mwakirunge dumpsite in Mombasa, Kenya. A total of 16 soil samples were collected using a randomized block design. The samples were inoculated in enriched basal media containing mixed municipal solid waste and incubated at 37°C for 21 days. Microbial identification was conducted using standard morphological, biochemical, and molecular approaches. DNA was extracted using organic isolation methods, and PCR amplification of the 16S rRNA gene for bacteria and the ITS gene for fungi was performed. Phylogenetic analysis grouped bacterial isolates into phylum Bacillota (Firmicutes), Pseudomonadota (Proteobacteria), and Actinomycetota (Actinobacteria) that included members of the genera bacilli, *Pseudomonas*, *brevibacilli*, *Microbacterium*, *Ochrobactrum*, *Paenibacillus*, *Staphylococcus*, *Isoptericola*, and *Streptomyces*. Fungal isolates belonged to the genus *Aspergillus* within the phylum Ascomycota. Three bacterial isolates B4S2 b (MZ571886), B3S1 (MZ571907), and B3S4 B (MZ571915) and one fungal isolate B2S2 a1 (MZ569413) had low sequence similarities with their closely known taxonomic relatives. The ability of the isolates to produce lipase, esterase, cellulase, amylase, and gelatinase enzymes was tested using the agar diffusion method. The results showed a significant level of enzyme production (*p* < 0.05). *Bacillus cereus* (MZ571899) exhibited the highest esterase activity; *Streptomyces thermocarboxydus* (MZ571882) exhibited the highest lipase activity, *Bacillus subtilis* (MZ571887) exhibited the highest amylase activity, and *Bacillus licheniformis* (MZ571888) exhibited the highest cellulase activity, while *Pseudomonas stutzeri* (MZ571900) exhibited the highest gelatinase activity. We recommend further studies to characterize the isolates with low sequence percentage similarities to establish their true identities. In addition, further enzymatic studies are required to quantify, characterize, and purify these enzymes for industrial applications.

## 1. Introduction

The menace of solid waste disposal is a widespread problem affecting both developed and developing countries [1]. Improper disposal strategies lead to environmental degradation and the proliferation of disease and generally affect livelihoods [2, 3]. In many developing countries, MSW management is an ignored part of the whole environmental management plan [4, 5]. In such countries, open dumping and open burning are still being practiced because of a lack of knowledge of the associated health risks, inadequate resources, and lack of political determination to guard and ameliorate environmental and public health [6]). Composting, waste-to-energy conversion, recycling, incineration, and landfilling are common methods of solid waste disposal. Incineration is a good method because the wastes are completely burnt down, but it can lead to the production of heavy toxic fumes of greenhouse gases like dioxins and furans [7]. Recycling provides an environmentally attractive solution. However, it is relatively ineffective and may decrease the quality of the resulting material. The process is expensive and may emit toxic compounds [8, 9]. The new products may also contain higher levels of foreign materials like colorants and additives which may alter the quality of the secondary material [10]. Landfilling is an ancient and simple method but also has limitations like land occupation and groundwater contamination [11–14].

Bioremediation is a more efficient and environmentally friendly approach that involves the use of plants, microorganisms, and their enzymes to break down environmental pollutants [15]. A variety of enzymes are produced by fungi and bacteria that can be useful in the degradation and detoxification of a wide range of wastes [16]. These enzymes include hydrolases such as lipases, proteases, DNases, pullulanases, xylanases, cellulases, amylases, phosphatases, and esterases among other enzymes [17, 18]. Hydrolases disrupt the major chemical bonds such as peptide bonds, esters, and carbon–halide bonds in pollutants, reducing them into less harmful products like water and carbon (IV) oxide [16, 19]. Microbial enzymes are useful in the degradation of environmental pollutants due to their high specificity to a wide range of substrates, tolerance to extreme conditions, high effectiveness at low concentrations of pollutants, and high mobility compared to microorganisms [20, 21]. Hydrolytic enzymes are important in the degradation of industrial wastes, plastic wastes, oil-contaminated soils, agro-based wastes, etc.

They have also found immense importance in industry, especially in the chemical, feed additive, biomedical science, and food industries [17, 22]. Different studies have investigated the ability of fungi to degrade wastes including kitchen wastes, plastic waste, and agricultural wastes among other waste materials. Some of the fungal species that have found importance in waste degradation include those from genera *Aspergillus*, *Fusarium*, *Penicillium, Trichoderma*, *Humicola, Mucor* sp., *Saccharomyces*, and *Candida* [23–27]. Some of the bacterial species that have commonly been associated with solid waste biodegradation include *Pseudomonas* sp., *Bacillus* sp., *Acetobacter*, *Lactobacillus* sp., *Streptococcus* sp., *Staphylococcus* sp., and *Actinobacteria*. Members belonging to *Actinobacteria* genera that have been demonstrated to play a role in waste biodegradation include *Micrococcus*, *Thermobifida*, *Streptomyces*, *Amycolatopsis*, *Thermobispora*, *Planomonospora*, *Cellulosimicrobium*, *Saccharopolyspora*, *Microbispora, Micromonospora*, *and Thermomonospora* [23, 28–30]. The aim of this study was to isolate and characterize potential waste-degrading microorganisms from the Mwakirunge dumpsite in Mombasa, Kenya.

## 2. Materials and Methods

### 2.1. Ethical Statement

The National Commission for Science, Technology and Innovation of Kenya (NACOSTI) approved this research study (License No: NACOSTI/P/23/25629). The field studies neither involved endangered nor protected species.

### 2.2. Study Site

Soil samples were collected from Mwakirunge dumpsite (3°57⁣′0⁣^″^ South, 39°40⁣′0⁣^″^ East), in Mombasa County, Kenya. Mwakirunge is the main official dumpsite for Mombasa wastes that was started in the year 2002. The dumpsite is about 15 km from the city center and is located along the flight path for Moi International Airport within the 13 km radius of the airport. The sampling points were randomized as follows: Block 1 (3°56⁣′27.0⁣^″^ S 39°40⁣′35.5⁣^″^ E), Block 2 (3°56⁣′27.4⁣^″^ S 39°40⁣′34.6⁣^″^ E), Block 3 (−3.941658, 39.672125), and Block 4 (−3.941414, 39.673460).

### 2.3. Sample Collection

Soil sampling was done using the randomized block design. About 100 g of soil mixed with solid wastes was collected from the dumpsite. The samples were collected from randomly selected blocks. From each block, sampling was done in four randomly selected points approximately 1.5 m apart, giving a total of 16 samples. Soil samples mixed with solid waste materials were collected by scooping at 5 cm depth in sterile Ziploc bags, labelled, and transported to the Kenya Marine and Fisheries Research Institute (KMFRI), where they were stored in the laboratory at 4°C for further analysis.

### 2.4. Microbial Culture Enrichment

Basal media was prepared following the protocol previously described by [31]. The media was prepared by dissolving (K_2_HPO_4_ 1 g; KH_2_PO_4_ 0.2 g; NaCl 1 g, MgSO_4_ 0.01 g; (NH_4_)_2_ SO_4_ 1 g; CaCl_2_.2H_2_O 0.002 g; FeSO_4_.7H_2_O 0.002 g) in 1 L of distilled water and enriched with mixed municipal solid wastes (paper waste, kitchen waste, and maize cobs sliced into small pieces of approximately 1 × 5 cm) that was then sterilized for 15 min at 121°C and 15 psi. Inocula were prepared following the procedure previously described by [24], where 2 g of soil was suspended in 100 mL of 0.85% sterile normal saline. Inoculation was done by adding 1% of the prepared inocula into 100 mL of basal media in a 250-mL Erlenmeyer flask. The negative control contained an uninoculated flask with basal media and waste materials. Incubation was done at 37°C and 130 rpm for 21 days in an orbital shaker. Microbial isolation was done from the enriched cultures in the incubation flasks after 21 days.

### 2.5. Isolation of Potential Useful Microorganisms

For bacterial cultures, a loopful of culture was spread using a sterile glass spreader on nutrient agar plates and incubated at 37°C for 24 h or up to 1 week for slow-growing bacteria. Pure cultures were obtained by continuously subculturing the mixed cultures on nutrient agar using the same conditions. For fungal cultures, a loopful of culture from the incubation flask was spread using a sterile spreader on potato dextrose agar and incubated at 37°C for a week. Pure fungal cultures were obtained by continuous subculturing of the obtained mixed cultures in PDA at 37°C for a week [24]. Actinomycetes were isolated by spreading a loopful of culture from the incubation flask on starch casein agar (SCA). The plates were incubated at 37°C for a week. Pure actinomycete colonies were obtained by continuous subculturing on SCA using the same conditions [32].

### 2.6. Characterization of Bacterial and Fungal Isolates

Identification of bacterial isolates was done based on their morphological, biochemical, and molecular characteristics. Colony morphology characterization was conducted using standard microbiological criteria with special emphasis on pigmentation, shape, form, elevation, and margin formation [33]. Physiological and biochemical analysis was determined using the following tests; Gram staining was done using the procedure described by [34]. Gram-positive organisms appear blue to purple while Gram-negative bacteria appear red to pink [34]. The ability of the bacterial isolates to produce indole from the degradation of tryptophan by the help of the tryptophanase enzyme was tested, and the formation of a red layer at the top of the tubes was an indication of a positive reaction [35]. The ability of the isolates to secrete enzyme catalase, which is responsible for the breakdown of hydrogen peroxide, was tested, and the formation of bubbles resulting from hydrogen peroxide breakdown to water and oxygen was an indication of a positive reaction [36]. The citrate utilization test was carried out as described by [37]. The bacterial growth and formation of a Prussian blue color indicated a positive reaction, while a negative result had no growth of bacteria and remained green. The methyl red (MR) test was done to determine the ability of bacteria to produce mixed acid products resulting from glucose fermentation while the Voges–Proskauer (VP) test was carried out to determine the ability of isolates to produce acetoin, a precursor to 2, 3 butanediol synthesis [38]. Lactophenol cotton blue staining of the fungal isolates was conducted as described by [39], and the presence of fungal hyphae, spores, conidia, and conidiophores was observed under the light microscope.

### 2.7. Screening Isolates for the Production of Extracellular Enzymes

The following enzymatic assays were conducted in triplicates. Semiquantitative tests for enzymatic (esterase, lipase, amylase, cellulase, and gelatinase) activities were carried out using techniques as previously described. Briefly, the media for the detection of esterase and lipase (on Tween 20 and Tween 80, respectively) were prepared as reported by [40, 41]. The culture plates were then observed for the appearance of clear zones or the presence of a precipitate around the colonies that indicated a positive result [40, 41]. The detection of amylase was prepared as described by [42]. The plates were then observed for the appearance of clear zones/decolorized regions around the colonies after flooding with Gram's iodine solution [42]. For the detection of cellulase and gelatinase activities, the procedures described by [43, 44] were used, respectively. Spot inoculation of test organism at the center of the media plates was done followed by incubation at 37°C for 2 days for the bacterial cultures and up to 5 days for fungal isolates. For gelatinase activity, the plates were flooded with saturated aqueous ammonium sulfate solution (NH_4_)_2_SO_4_) and then observed for halo zones around colonies after removing the excess solution [44], while for cellulase activity, the culture plates were flooded with 0.1% Congo red dye for 15 min followed by flooding with 1 M NaCl. This was discarded after 15 min after which the plates were observed for clearance zones around colonies [43].

### 2.8. DNA Extraction and PCR Amplification of the 16S rRNA Gene Region

Representative bacterial isolates (30) were selected based on their unique morphological characters for molecular characterization. The isolates were cultured in 25 mL of LB broth in 50 mL flasks for 48 h at 37°C and 130 rpm. The cultures were mixed by shaking and centrifuged in 5 mL falcon tubes at 4500 rpm for 10 min. The supernatant was discarded, and the pellet was retained for DNA extraction as described by [45]. The recovered pure DNA was confirmed by gel electrophoresis using 1% agarose in 1× TAE buffer and viewed under ultraviolet after staining with Sybr Green [45]. PCR was done in a Genesy Thermal Cycler machine (China) using prokaryotic universal primer pair 27F (5⁣′-AGAGTTTGATCMTGGCTCAG-3⁣′) and 1492R (5⁣′-CGGTTACCT TGTTACGACTT-3⁣′) [46]. The PCR was conducted in a reaction mixture containing 10 *μ*L of NEB OneTaq 2X Master Mix with Standard Buffer (BioLabs), 1 *μ*L genomic DNA, 1 *μ*L forward primer (10 *μ*M), 1 *μ*L reverse primer (10 *μ*M), and 7 *μ*L of nuclease-free water (Catalogue No. E476). The PCR was done using the following conditions: initial denaturation at 94°C for 5 min followed by 35 cycles of denaturation at 94°C for 30 s, annealing at 50°C for 30 s, and extension at 72°C for 1 min, followed by final extension at 72°C for 10 min. The integrity of the PCR amplicons was confirmed by visualization on a 1% agarose gel (CSL-AG500, Cleaver Scientific Ltd) stained with EZ-vision Bluelight DNA Dye (AMRESCO, LLC) under a gel documentation chamber.

### 2.9. DNA Extraction and PCR Amplification of the ITS Gene Region

Fungal mycelia grown for 4 days at 37°C on potato dextrose agar were cut using a sterile scalpel, then frozen in liquid nitrogen, and crushed with the help of a sterile pestle and mortar. The crushed mycelia were then used for DNA extraction following the method previously described by Aamir et al. (2015). Quality of the extracted DNA was checked through gel electrophoresis using 0.8% agarose gel in 1× TAE buffer and viewed under ultraviolet by staining with Sybr Green (Thermo Fisher Scientific). The extracted DNA was stored at −20°C awaiting further applications.

Amplification of the fungal (ITS) regions was done using ITS1F (5⁣′CTTGGTCATTTAGAGGAAGTAA3⁣′) and ITS4R (5⁣′TCCTCCGCTTATGC 3⁣′) primer sets [47]. Amplification was carried out in a 0.2 mL tube and reaction mixture containing 10 *μ*L of NEB OneTaq 2X Master Mix with Standard Buffer (Catalogue No. M0482S), 1 *μ*L Genomic DNA (30-80 ng/*μ*L), 1 *μ*L forward primer (10 *μ*M), 1 *μ*L reverse primer (10 *μ*M), and 7 *μ*L of nuclease-free water (Catalogue No. E476). The PCR was done in Genesy Thermal Cycler machine (China) using the following conditions: initial denaturation at 94°C for 3 min followed by 30 cycles of denaturation at 94°C for 1 min, annealing at 55°C for 1 min, initial extension at 72°C for 3, and final extension at 72°C for 10 min. The quality of the PCR amplicons was confirmed by visualization on a 1% agarose gel (CSL-AG500, Cleaver Scientific Ltd) stained with EZ-vision Bluelight DNA Dye (AMRESCO, LLC) under a gel documentation chamber.

### 2.10. Purification and Sequencing of PCR Products

Purification of all the PCR amplicons was done using ExoSAP-IT PCR Product Cleanup reagent (Thermo Fisher Scientific) by following the instructions of the manufacturer. Briefly, the Exo/SAP master mix was prepared by adding the following to a 0.6-mL microcentrifuge tube: 50 *μ*L Exonuclease I (20 U/ul) and 200 *μ*L shrimp alkaline phosphatase (1 U/uL). The reaction mixture was prepared by adding 10 *μ*L of the PCR amplicons to 2.5 *μ*L of the previously prepared ExoSAP Mix. These were mixed well and then incubated at 37°C for 15 min. The mixture was then heated at 80°C for 15 min to stop the reaction. The purified amplicons were packaged and sent for sequencing by a commercial service provider at Inqaba Biotech (South Africa).

### 2.11. Phylogenetic Analysis

Sequences of the isolates were manually edited in chromas (http://www.technelysium.com.au/ChromasPro.html) and checked for the presence of artifacts or sequencing errors using Mallard software [48], an NCBI bioinformatic tool for detecting chimera sequences. A search for similar sequences using BLASTN [49] was performed, and sequence alignment was performed using the CLUSTAL Omega program (http://www.clustal.org) against the nearest neighbors. A neighbor-joining tree of the aligned sequences was constructed [50] using MEGA X software [51]. Evolutionary distances were computed using the maximum composite likelihood method [52]. To obtain statistical support values for the branches, bootstrapping [53] was conducted with 1000 replicates. All sites, including gaps in the sequence alignment, were excluded pairwise in the phylogenetic analysis. Using the resultant neighbor-joining tree, each isolate was assigned to the proper taxonomic group. The taxonomic assignment was confirmed at a 95% confidence level using the RDP Naïve Bayesian rRNA Classifier Version 2.11 on the RDP website [54].

## 3. Results

### 3.1. Morphological Characters of Bacterial Isolates

**Figure 1:**
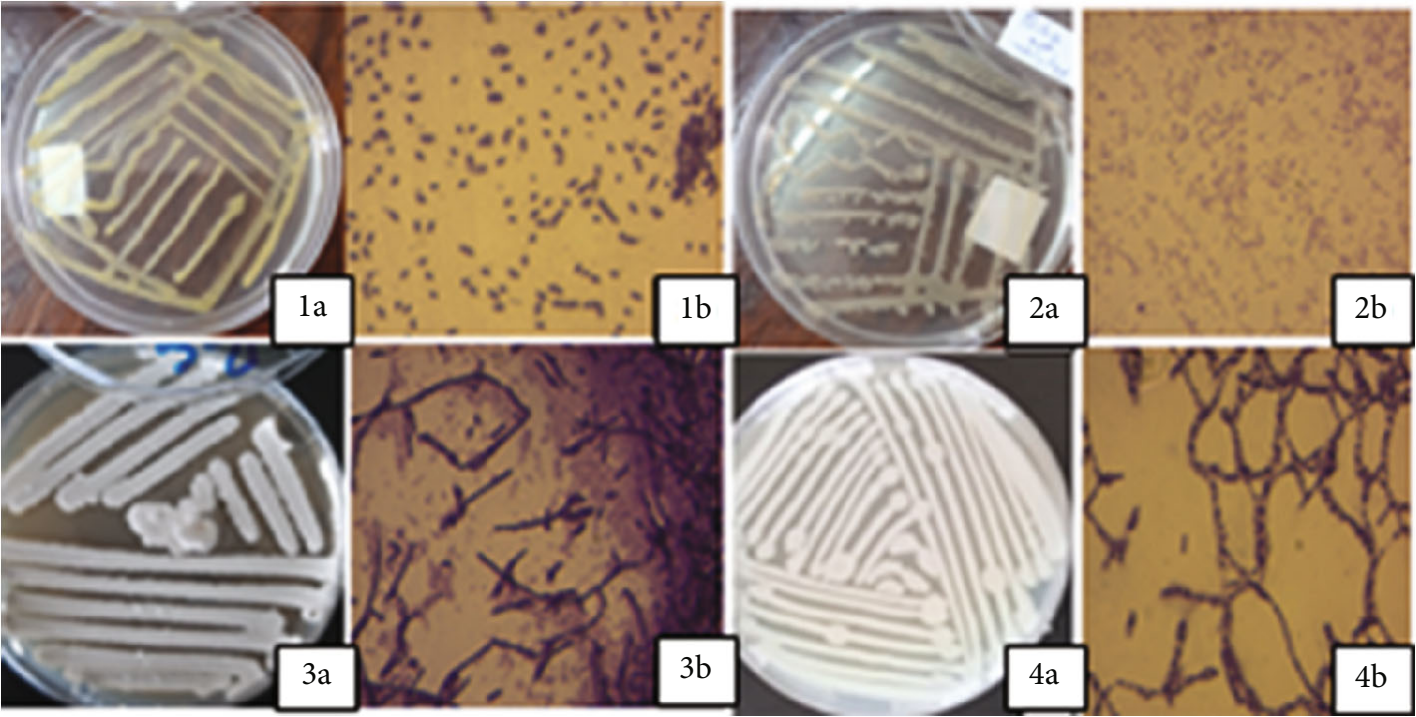
Plate cultures and Gram staining results of some representative bacterial isolates. (1a–4a) Representatives of bacterial culture plates; (1b) Gram-positive cocci; (2b) Gram-negative cocci; (3b, 4b) Gram-positive rods.

The purified bacterial colonies showed differences in their surfaces, forms, optical characteristics, margins, elevations, and color. About 70% of the colonies were cream, entire, and smooth in nature. The majority of the bacterial isolates (77%) were Gram-positive, out of which 70% were rod shaped and 7% were cocci. The rest (33%) were Gram-negative rods (Figure 1, Table 1).

### 3.2. Biochemical Characters of Bacterial Isolates

The isolated bacteria were tested for indole production, citrate utilization, catalase production, and MR-VP tests. All the isolated bacteria were catalase-positive except isolates B2S4b, B4S1 A3, and B3S4 A2. All the isolates were citrate positive except isolates B3S2 b, B2S3 b1, B3S4 b3, B1S3 A, B4S1 A3, and B3S4 A2. All the isolates were incapable of producing enzyme tryptophanase which degrades tryptophan to produce indole. Thirteen isolates (B3S4 A2, B3S4 A1, B3S4 b1, B2S4 A, B2S3 b1, B3S3 1, B3S3 2, B3S2 a, B3S1, B3S3 b2, B2S2, B4S4 A1, and B1S2) were MR positive, indicating glucose fermentation ability of the isolates that was characterized by red color upon addition of three drops of MR indicator. All the isolates tested negative for VP (Table 1**).**

### 3.3. Morphological Characters of Fungal Isolates

**Figure 2:**
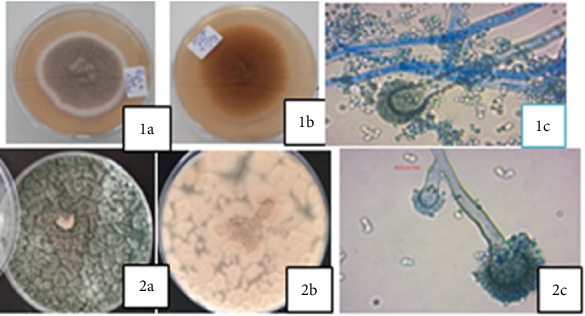
Plate culture and microscopic features of some representative fungal isolates. (1a, 2a; 1b, 2b) The 3-day-old plate cultures on the front and reverse sides, respectively. (1c, 2c) The microscopic features of the respective fungal representatives.

A total of 20 fungi were successfully isolated on PDA, and 80% of the isolated fungi were greenish to gray on the front view and cream-white on the reverse side. These isolates also had a characteristic white border encircling the mycelia. The colonies showed good sporulation and fast growth at 37°C. Microscopically, the conidia, conidiophores, and the hyphae were clearly seen on lactophenol cotton blue staining (Table 2; Figure 2). All the isolates showed erect conidia, branching-septate hyphae, and club-shaped vesicles. A foot cell producing a single conidiophore perpendicular to the long axis of the cell characteristic to the genus *Aspergillus* was also observed. All the fungal isolates were classified based on their macro and microscopic characteristics as members of the genus *Aspergillus* (Table 2; Figure 2).

### 3.4. Affiliations of Bacterial Sequences With Closely Related Taxonomic Relatives

PCR amplification of the 16S rRNA gene region for the bacterial isolates (A1-A8, B1-B22) was successful. Sequence similarity search at the nucleotide sequence database (GenBank) indicated close taxonomic affiliation of the newly obtained sequences with known cultivatable members of different genera belonging to the phyla Actinobacteria, Firmicutes, and Proteobacteria. The 16S rRNA gene sequences of the majority of the isolates had close sequence similarities (97-100%) with known bacterial species isolated from the different habitats, except for isolates B4S2b (MZ571886), B3S1 (MZ571907), and B3S4B (MZ571915) that had sequence similarities with known taxonomic relatives of 93%, 92% and 92%, respectively (Table 3).

Thirteen isolates were associated with the genus *Bacillus* with sequence identities of 99%–100%, except for isolate B4S2b (MZ571886), which had 93% sequence similarity against *B. sonorensis* (Table 3). Four isolates (B3S4 b1 (MZ571896), B3S2 b (MZ571900), B4S1 A3 (MZ571909), and B2S1 (MZ571910)) were closely affiliated with members of the genus *Pseudomonas* with sequence identities of between 99% and 100% (Table 3). Isolate B2S2b2 (MZ571882) was closely affiliated to *Streptomyces thermocarboxydus* (KJ018992) with a sequence similarity value of 100%. Isolates B3S4b (MZ571913) and B4S4A1 (MZ571911) had 99%–100% sequence identities with members from the genera *Paenibacillus*and *Pseudoxanthomonas,* respectively. Two isolates (B1S4b (MZ571898) and B3S1 (MZ571907) had sequence similarities of 100% and 92%, respectively, with members of the genus *Ochrobactrum*. Other two isolates B1S2 (MZ571912) and B3S4A2 (MZ571914) were affiliated with members from the genus *Microbacteria* with sequence similarity of 99%. The other isolates B3S4F (MZ571897) and B3S3B (MZ571884) were affiliated (with 99%–100% sequence similarities) to members from the genera *brevibacilli* and *Isoptericola*, respectively (Table 3).

### 3.5. Phylogenetic Analysis of Bacterial Isolates

**Figure 3:**
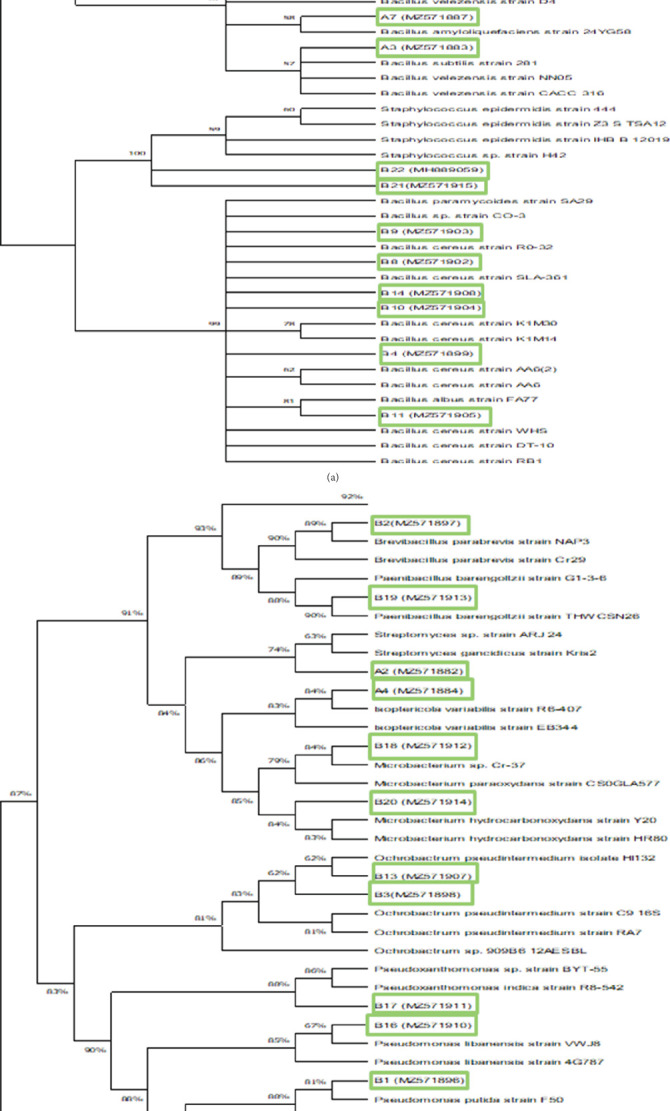
(a) Consensus phylogenetic tree based on partial bacterial 16S rRNA sequences, inferred by using the maximum likelihood method on Mega X. (b) Consensus phylogenetic tree based on partial bacterial 16S rRNA sequences, inferred by using the maximum likelihood method on MEGAX.

All the analyzed bacterial sequences were deposited at the primary nucleotide sequence database (GenBank) with the accession numbers (MZ571881-MZ571916). The consensus evolutionary tree constructed based on partial bacterial 16S rRNA sequences grouped the isolates into three major clusters constituting members of the phyla Bacillota (Firmicutes), Pseudomonadota (Proteobacteria), and Actinomycetota (Actinobacteria) (Figure 3a,b). The cluster associated with the phylum Firmicutes included members of the genera *Bacillus* and *Staphylococcus* with bootstrap support values of between 99% and 100%. This major cluster had four subclusters constituting species of *S. epidermidis*, *B. paralicheniformis*, *B. licheniformis*, *B. sonorensis*, *B. velezensis*, *B. subtilis*, *B. cereus*, *B. albus*, and *B. paramycoides* (Figure 3a). The other major cluster was represented by members from the phylum Actinobacteria and included members of the genera *Microbacterium, Streptomyces, Isoptericola*, and *Brevibacillus* with a bootstrap support value of 91%. The last cluster constituted bacterial members from the phylum Proteobacteria that included members of the genera *Ochrobactrum*, *Pseudomonas*, and *Pseudoxanthomonas* with a bootstrap support value of 83% (Figure 3b). An Archaea *Vulcanisaeta disttibuta (*NR_040876.1) was used as an outgroup to root the tree.

### 3.6. Affiliations of Fungal Sequences With Closely Related Taxonomic Relatives

The PCR amplification of the ITS gene region of the 15 fungal isolates using universal primers ITS1F and ITS4R was successful. Taxonomic analysis of sequences of the isolates showed the presence of cultivatable members of the genus *Aspergillus* in the phylum Ascomycota. ITS gene sequences of the isolates indicated high similarity (100%) with sequences of several members of the genus *Aspergillus* (*A. niger*, *A. nidulans*, *A. terreus*, and *A. fumigatus*), except isolate B2S2 a1 (MZ569413), which had a percentage sequence similarity of 96% (Table 4).

### 3.7. Phylogenetic Analysis of Fungal Isolates

**Figure 4:**
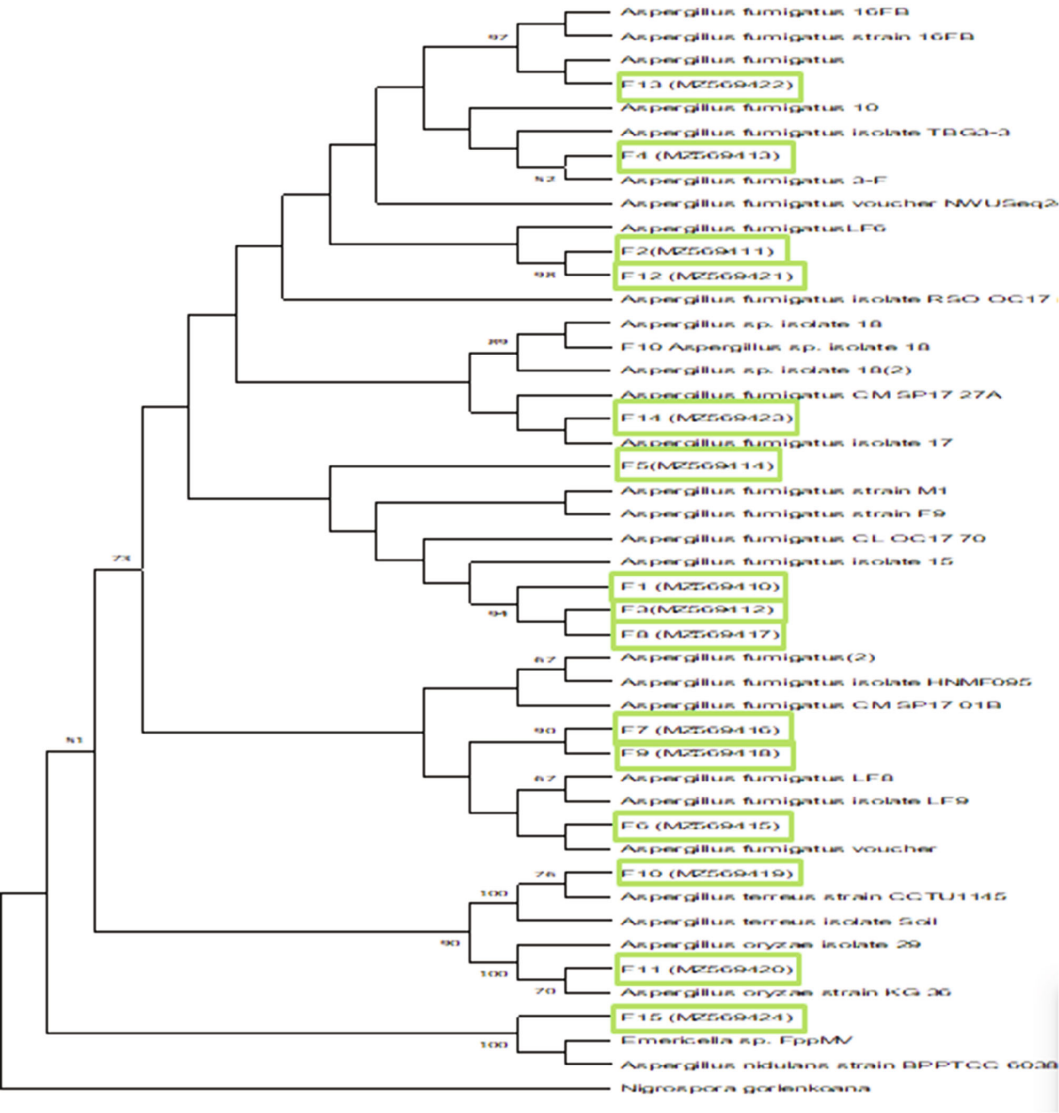
Consensus phylogenetic tree based on ITS gene regions of the fungal isolates, inferred by using the maximum likelihood method. *Nigrospora gorlenkoana* is an out-group used to root the tree.

The analyzed ITS gene sequences were deposited at the primary nucleotide sequence database (GenBank) with the accession numbers (MZ569410-MZ569424). The inferred phylogenetic tree based on partial ITS gene region sequences grouped the isolates into one major cluster comprising members of the genus *Aspergillus* in the phylum Ascomycota. The subclusters were represented by species such as *A. fumigatus*, *A. tereus*, *A. oryzae*, *and A nidulans* (Figure 4). *A. fumigatus* formed the majority of the members with 100% sequence similarity with known taxonomical relatives (Figure 4 and Table 4).

### 3.8. Extracellular Enzyme Production From Bacterial Isolates

Microbial selection for enzymatic activity was based on preliminary enzymatic assay results. Isolates that showed good activity during preliminary screening were selected for further screening. Ten fungal and 16 bacterial isolates were selected and screened for their potential to secrete useful enzymes. Among the tested bacterial isolates, 11 isolates exhibited amylolytic activity, while cellulase activity was observed in 10 isolates. Only four isolates including *Streptomyces thermocarboxydus* A2*, Bacillus cereus* B4*, Pseudomonas stutzeri* B5, and *Paenibacillus barengoltzii* B19 exhibited lipolytic activity on Tween 80. Additionally, nine isolates exhibited esterase activity.

Isolate B2S2 b2-*Streptomyces thermocarboxydus* (MZ571882) tested positive for all the screened enzymes and had a significantly high lipase activity (23.33 ± 1.76 mm) (*p* < 0.05). Isolates B3S4 A1-*Bacillus* sp. A5- (MZ571885), B3S3 2- *Bacillus* sp. B9 (MZ571903), and B1S3 A- *Bacillus* sp. B12 (MZ571906) tested positive for amylase and cellulase enzymes (Table 5). B2S1 A2-*Bacillus* sp. (MZ571887) (25 ± 5.13 mm) and B1S3 A*-Bacillus* sp. (MZ571906) (23.67 ± 0.88 mm) had a significantly high amylase activity (*p* < 0.05), while B2S2 b-*Bacillus licheniformis (*MZ571888) (69 ± 2.65 mm) and B3S4 A2-*Bacillus* sp. (MZ571881) (46.67 ± 5.23 mm) exhibited the highest cellulase activity which was significantly different from all the other isolates (*p* < 0.05). B2S1 A2-*Bacillus* sp. (MZ571887) showed some activities for enzymes amylase (25.00 ± 5.13 mm), cellulase (27.33 ± 0.67 mm), and esterase (18.67 ± 0.67 mm). Isolate B3S1-*Ochrobactrum* sp. (MZ571907) also showed activity for amylase (13.00 ± 1.15 mm), cellulase (28.00 ± 4.16 mm), and esterase (16.33*d* ± 0.88 mm) (Table 2). Both B2S1 A2-*Bacillus* sp. (MZ571887) and B3S1-*Ochrobactrum* sp. (MZ571907) did not show activities for enzymes lipase and gelatinase. B2S4 A*-Bacillus cereus* (MZ571899) showed some activities for esterase (72.67 ± 1.76 mm), lipase (14.33 ± 1.20 mm), and gelatinase (22.00*b* ± 2.52 mm) enzymes and no activity for both amylase and cellulase enzymes. It showed the highest esterase activity (72.67 ± 1.76 mm), which was significantly different from all the isolates screened. Isolate B3S2 b- *Pseudomonas stutzeri (*MZ571900) tested positive for all the enzymes screened except for cellulase and had a significantly high gelatinase activity of (34.67 ± 1 mm) (*p* < 0.05). Isolates B3S3 1-*Bacillus* sp. (MZ571902) B8 and B4S4 B1-*Bacillus* sp. A3 (MZ571883) had positive enzyme activities for all the screened enzymes except for lipase. Isolate B4S4 B1 (MZ571883) showed significantly high cellulase (37.00 ± 1.53 mm) and esterase activities (53.00 ± 1.15 mm). *Bacillus albus* B11 (B3S2a) and (MZ571905) tested positive for enzymes amylase (20.00 ± 1.15), cellulase (12.67 ± 1.45 mm), and gelatinase (13.00 ± 1.15 mm). B4S4 A1-*Pseudoxanthomonas* sp. B17 (MZ571911) showed activity for only two enzymes: amylase (7.33 ± 1.33 mm) and gelatinase (12.00 ± 1.00 mm). B3S4 A2 *Bacillus* sp. A1-(MZ571881) and B2S2 b-*Bacillus* sp. A8 (MZ571888) tested negative for all the enzymes screened except cellulase. *Bacillus* sp. A8 had the highest cellulase activity (69 ± 2.65 mm), which was significantly different at (*p* < 0.05) among all the tested isolates. B3S4 A2-*Microbacterium* sp. B20 (MZ571914) was positive for esterase enzyme (15.33 ± 1.76 mm) only.

### 3.9. Extracellular Enzyme Production From Fungal Isolates

Ten fungal isolates were qualitatively screened for their potential to secrete enzymes cellulase, lipase, amylase, esterase, and gelatinase. The growth of fungal isolates on the plates was uneven, and therefore, the diameter zone of clearance could not be accurately measured. Low, moderate, and high activities for amylase, cellulase, and gelatinase were detected for some isolates as shown in Table 6.

No esterase and lipase activities were recorded in all the screened fungal isolates. Among the 10 isolates tested, *Aspergillus niger* F6 (MZ569415) did not exhibit amylolytic activity. Nine isolates tested positive for cellulase activity except *Aspergillus nidulans–*F15 (MZ569424). Eight isolates exhibited gelatinolytic activity except two isolates *Aspergillus terreus* F10 (MZ569419) and *Aspergillus fumigatus* F13 (MZ569422).

## 4. Discussion

The occurrence and diversity of potentially useful microorganisms vary with environments and geographical locations. Microorganisms interact with waste materials (pollutants) and secrete extracellular enzymes, which catalyze the degradation of the pollutants primarily through oxidative reactions or hydrolytic activity. The ability of the microbes to produce extracellular enzymes indicates their possible roles in waste biodegradation and other biotechnological applications. In this study, microorganisms were isolated after a culture enrichment period that allowed microbes to thrive on media supplemented with mixed municipal solid waste materials.

Phylogenetic analyses of the 16S rRNA gene region in the current study categorized the majority of the isolates (with 47% frequency) as members of the genus *Bacillus* (Table 3). About 50% of *Bacillus* species were capable of multiple enzyme production, indicating their possible implications in municipal solid waste degradation. Notably, bacilli species are capable of producing thick-walled endospores resistant to heat, radiation, and other harsh environmental conditions which may be the reason for their frequent occurrence [24]. This is consistent with the studies conducted by [24, 55] where *Bacillus* species were among the most frequently isolated soil bacteria. Some members from this genus have ecological significance including implications in nutrient cycling, improvement of stress tolerance to plants, and in waste degradation [18]. *Bacillus* species also have potential for industrial applications due to their ability to secrete extracellular enzymes. They are an affordable source of enzymes because they are widely distributed, they are safe to work with, they are easy to cultivate, and they are susceptible to genetic transformations [56] and have found importance in the agricultural, biomedical, feed and additive, food, and the chemical industry [49–52].

In the present study, a number of bacilli sp. demonstrated esterase and lipase activities. Production of lipase and esterase enzymes by these isolates suggests their potential to degrade oil-based waste materials and even plastic wastes [41, 57–61]. These results are in agreement with other studies, where *Bacillus* species have been reported to produce lipolytic enzymes [34, 55, 56, 62]. Moreover, some *Bacillus* isolates indicated cellulase activity that is consistent with the results reported by [57, 58]. The production of cellulases by the isolates suggest their importance in plant-biomass/cellulosic waste degradation [63, 64]. It should be noted that municipal solid wastes constitute about 40%–50% of cellulose, which provides a good environment for the growth of cellulose-degrading microorganisms [26].

*Pseudomonas stutzeri* B5 (MZ571900) isolated from this study had the highest protease activity and was capable of producing multiple enzymes. The multiple enzyme secretion indicates its possible role in the degradation of a variety of wastes. *Pseudomonas* species have often been implicated in the decomposition of organic matter degradation of plastic waste and wastewater degradation among other waste [65–67]. Good enzyme production abilities by both bacilli and *Pseudomonas* species indicate their significant role in waste decomposition. Also, *Streptomyces thermocarboxydus* had activity for all the screened enzymes (Table 5). High diversity of enzymes in *Streptomyces* spp. was also reported by [68]. A lipase-producing *Streptomyces* sp. was reported by [69] while a cellulolytic *Streptomyces* sp. was isolated from composting materials by [70]. Members of the phylum actinobacteria have been associated with the secretion of many useful bioactive compounds and enzymes [71, 72].

Analysis of the fungal ITS gene regions in this study indicated that 73% of the isolates were associated with *Aspergillus fumigatus* (Table 4). These results are in agreement with previous studies conducted by [73, 74]), where *A. fumigatus* was reported as the most common fungal species in waste and compost. *A. fumigatus* is, however, known to be the most prevalent airborne fungal pathogen that may cause aspergillosis and other lung infections, especially in immunosuppressed individuals. The abundant development of *A. fumigatus* in waste and compost is, therefore, a matter of concern due to their potential of causing disease. The consistent exposure and inhalation of fungal particles pose a risk of aspergillosis and other lung infections among the people living and working in the waste disposal areas [75]. *Aspergillus* species including *A. flavus*, *A. terreus*, *A. fumigatus*, and *A. niger* were isolated from compost by [74]. *Aspergillus niger*, *A. flavus*, and *A. terreus* capable of decomposing green household waste were also isolated by [76]. The ability of these fungal isolates to produce extracellular hydrolases including cellulase, amylase, and gelatinase is of ecological importance. Contrary to other studies, no lipase nor esterase activity was detected in all the fungal isolates screened. Previous studies have, however, reported lipolytic activity in *Aspergillus* species [77–80]. All the isolates screened were cellulase-active except isolate F15- *A. nidulans (*MZ569424). Cellulase production in *Aspergillus* species has been widely reported in previous studies. In a study by [81], two *Aspergillus* species including *A. niger* and *A. flavus* isolated from waste dumpsite were reported to have cellulase activity. Amylase production in *A. niger* has been described by several researchers [82–84], whereas amylase production in *Aspergillus fumigatus* was reported by [85]. Amylase production by *Aspergillus* species implicates them in the degradation of starch-based waste and also their importance in industrial applications. Protease production in *Aspergillus* species has also been reported in previous studies. Saline-tolerant protease secreted by *Aspergillus* sp. isolated from soil was reported by [86]. In another study by [87], *A. oryzae*, commonly used in the manufacture of Chinese soy sauce, was reported to be capable of producing three types of extracellular proteases [88] and reported good protease activity in *A. flavus* and *A. fumigatus* isolated from a local rice husk dumpsite.

In this study, three bacterial isolates, B4S2 b (MZ571886), B3S1 (MZ571907), and B3S4 B (MZ571915), and one fungal isolate, B2S2 a1 (MZ569413), had low sequence percentage similarity based on the partial 16S rRNA gene region analysis. Therefore, a further polyphasic approach including analysis based on other housekeeping genes and/or full genome sequencing is required to establish the true identity of these isolates. This study corroborates that municipal solid waste dumpsite is a potential source for a wide spectrum of bacteria and fungi with the potential of producing a variety of enzymes that can have diverse industrial applications.

### 4.1. Study Limitations

It should be noted that all culture experiments were conducted under laboratory settings; hence, the growth conditions used in this study may have limited the growth of other microorganisms that require specific growth environments. Also, considering the paradigm that only 1% of microorganisms is culturable [89], such limitation affected this study.

The analysis of the 16S rRNA and ITS gene regions alone was not sufficient to declare the novelty of the isolates recovered from this study, which had a low percentage similarity with their closely known taxonomic relatives. Also, the study had fund limitations that could not allow us to perform specific experiments to demonstrate the degradation of specific waste materials.

## Figures and Tables

**Table 1:** Biochemical characters of the bacterial isolates.

**Isolates code**	**Accession no.**	**Gram reaction**	**Catalase**	**MR**	**Citrate**	**Indole**
B3S4 A2	MZ571881	GPR	+	+	+	−
B2S2 b2	MZ571882	GPR	+	−	+	−
B4S4 B1	MZ571883	GPR	+	−	+	−
B3S3 b	MZ571884	GPR	+	−	+	−
B3S4 A1	MZ571885	GPR	+	+	+	−
B4S2 b	MZ571886	GPR	+	−	+	−
B2S1 A2	MZ571887	GPR	+	−	+	−
B2S2 b	MZ571888	GPR	+	−	+	−
B3S4 b1	MZ571896	GNR	+	+	+	−
B3S4 f	MZ571897	GPR	+	−	+	−
B1S4 b	MZ571898	GPR	+	−	+	−
B2S4 A	MZ571899	GPR	+	+	+	−
B3S2 b	MZ571900	GNR	+	−	−	−
B2S3 b1	MZ571901	GPR	+	+	−	−
B3S3 1	MZ571902	GPR	+	+	+	−
B3S3 2	MZ571903	GPR	+	+	+	−
B3S4 b3	MZ571904	GPR	+	−	−	−
B3S2 a	MZ571905	GPR	+	+	+	−
B1S3 A	MZ571906	GPR	+	−	+	−
B3S1	MZ571907	GPR	+	+	+	−
B3S3 b2	MZ571908	GPR	+	+	+	−
B4S1 A3	MZ571909	GPR	−	−	+	−
B2S2	MZ571910	GNR	+	+	+	−
−B4S4 A1	MZ571911	GNR	+	+	−	−
B1S2	MZ571912	GNR	+	+	+	−
B3S4 b	MZ571913	GPR	+	+	+	−
B3S4 A2	MZ571913	GNR	−	−	−	−
B3S4 bs	MZ571915	GPC	+	+	−	−
B1S2 A1	MZ571916	GPC	+	−	−	−

*Note:* Key: (+) denotes positive; (−) denotes negative.

Abbreviations: GNR, Gram-negative rods; GPC, Gram-positive cocci; GPR, Gram-positive rods.

**Table 2:** Macroscopic and microscopic features of fungal isolates.

**Isolates code**	**Accession no.**	**Macroscopic features**	**Microscopic features**	**Reverse side**
B2S3	MZ569410	Greenish with white margin, fast growing, velvety	Branching hyphae, septate, and hyaline club-shaped conidiophore	Cream
B4S2	MZ569411	Greenish-gray, cottony, fast growing	Branching hyphae, septate, and hyaline club-shaped conidiophore	Cream
B3S4 b2	MZ569412	Grayish, fast growing, velvety	Branching hyphae, septate, and hyaline club-shaped conidiophore	Cream
B2S2 a1	MZ569413	Grayish, powdery surface, fast growing	Branching hyphae, septate, and hyaline club-shaped conidiophore	Cream
B3S2 a	MZ569414	Greenish with white margin, powdery texture, fast growing	Branching hyphae, septate, and hyaline club-shaped conidiophore	Cream
B3S3 b1	MZ569415	Grayish, powdery texture, fast growing	Branching hyphae, septate, and hyaline club-shaped conidiophore	Cream
B4S4 b	MZ569416	Greenish-gray, velvety, fast growing	Branching hyphae, septate, and hyaline club-shaped conidiophore	Cream
B3S2 b	MZ569417	Green with brown appearance at the center, fast growth, cottony	Branching hyphae, septate, and hyaline club-shaped conidiophore	Cream
B3S2 A	MZ569418	Green with white margin, fast growth, cottony	Branching hyphae, septate, and hyaline club-shaped conidiophore	Cream
B3S1 b	MZ569419	Brownish with white margin, wooly appearance, fast growing	Branching hyphae, septate, and hyaline club-shaped conidiophore	Brown
B3S3 b	MZ569420	Olive with shiny white margin, slow growing, velvety	Branching hyphae, septate, and hyaline club-shaped conidiophore	Brown
B4S2	MZ569421	Grayish with brown center, fast growing, powdery texture	Branching hyphae, septate, and hyaline club-shaped conidiophore	Cream
B3S4 b2	MZ569422	Greenish, velvety, fast growth	Branching hyphae, septate, and hyaline club-shaped conidiophore	Brown
B3S1 A	MZ569423	Brownish, powdery texture, fast growth	Branching hyphae, septate, and hyaline club-shaped conidiophore	Brown
B4S4	MZ569424	Surface green with white periphery, slow growing, wooly	Branching hyphae, septate, and hyaline club-shaped conidiophore	Cream

**Table 3:** Affiliation of bacterial isolates with their closest known taxonomic relatives.

**Seq**	**Isolates**	**Accession no.**	**Closest taxonomic relative**	**Source of affiliates**	**Country of isolation**	**% ID**
**ID**	**Code**					
AI	B3S4 A2	MZ571881	*Bacillus paralicheniformis* strain *CB211* (MT527538)	Soil	China	99
A2	B2S2 b2	MZ571882	*Streptomyces thermocarboxydus* strain *SDT64*(KJ018992)	Dark tea	China	100
A3	B4S4 B1	MZ571883	*Bacillus velezensis* strain *ROA048*(MT525304)	Sediment	South Korea	100
A4	B3S3 b	MZ571884	*Isoptericola* sp. strain *G235TR* (MZ182281)	Insects	Germany	99
A5	B3S4 A1	MZ571885	*Bacillus licheniformis* strain *I97(KU922426)*	Air	China	99
A6	B4S2 b	MZ571886	*Bacillus sonorensis* strain *JUG RS2(*MK156710)	Soil	India	93
A7	B2S1 A2	MZ571887	*Bacillus subtilis* strain *MGB4024* (MH261154)	Soil	China	100
A8	B2S2 b	MZ571888	*Bacillus licheniformis* strain *7B3-13*(MK603064)	Sediment	Brazil	99
B1	B3S4 b1	MZ571896	*Pseudomonas putida* strain *S4k76* (MK737354)	Soil	Iran	100
B2	B3S4 f	MZ571897	*Brevibacilli parabrevis* strain *GF13*(KY312740)	Intestinal tract	China	100
B3	B1S4 b	MZ571898	*Ochrobactrum pseudintermedium* (LR135573)	*Hermetia illucens*	Italy	100
B4	B2S4 A	MZ571899	*Bacillus cereus* strain *K1M36*(MW559327)	Air	China	100
B5	B3S2 b	MZ571900	*Pseudomonas stutzeri* strain *AAK_MD_30* (MT180597)	Mule dung	India	100
B7	B2S3 b1	MZ571901	*Bacillus velezensis* strain *LZ12-07*(MT845849)	Soil	China	100
B8	B3S3 1	MZ571902	*Bacillus cereus* strain *B236*(KF494193)	Feces of piglets	China	100
B9	B3S3 2	MZ571903	*Bacillus paramycoides* strain *2914* (MT611870)	—	China	100
B10	B3S4 b3	MZ571904	*Bacillus cereus* strain *XS 24-5*(MT000038)	—	Southeast China	100
B11	B3S2 a	MZ571905	*Bacillus albus* strain *FA26* (MK993444)	Soil	Pakistan	100
B12	B1S3 A	MZ571906	*Bacillus subtilis* subsp. *stercoris* strain *EGI143* (MN704442)	*Origanum vulgare* L.	China	100
B13	B3S1	MZ571907	*Ochrobactrum sp* strain *CSL1 16S*(MG008507)	—	China	92
B14	B3S3 b2	MZ571908	*Bacillus cereus* strain *XaM13* (MW559356)	—	China	100
B15	B4S1 A3	MZ571909	*Pseudomonas stutzeri* strain *W41* (KT380584)	Sediment	China	99
B16	B2S2	MZ571910	*Pseudomonas sp.* strain *CR7* (MN087814)	Glacier	China	99
B17	B4S4 A1	MZ571911	*Pseudoxanthomonas indica* strain *ES-4* (MK537384)	Soil	India	100
B18	B1S2	MZ571912	*Microbacterium paraoxydans* strain *SMV194#22*(KP780213)	—	Italy	99
B19	B3S4 b	MZ571913	*Paenibacillus barengoltzii* strain *THWCS8 (*GQ284355)	Sediment	India	99
B20	B3S4A2	MZ571914	*Microbacterium* sp. *HBUM178961* (KR906353)	Medicinal plants Gynura	China	99
B21	B3S4 B	MZ571915	*Staphylococcus epidermidis* strain *SSR4* (MH889059)	Soil	Peru	92
B22	B1S2 A1	MZ571916	*Staphylococcus epidermidis* strain *SESURV_p3*(CP043801)	—	United States	97

**Table 4:** Affiliation of ITS gene sequences of the isolates with their closest known taxonomic relatives.

**Isolates code**	**Accession no.**	**Closest taxonomic relative**	**Source**	**Country of isolation**	**% ID**
B2S3	MZ569410	*Aspergillus fumigatus* isolate *ZSY-5-4(*MT487775)	N/A	China	100
B4S2	MZ569411	*Aspergillus fumigatus* strain *N15-2-1-1 (*MN704714)	N/A	China	100
B3S4 b	MZ569412	*Aspergillus fumigatus* isolate *ZSY-5-4 (*MT487775)	N/A	China	100
B2S2 a1	MZ569413	*Aspergillus fumigatus* isolate *19-10-1*(HQ149777)	Marine environment	China	96
B3S2 a	MZ569414	*Aspergillus fumigatus* strain *ND108*(MH270614)	Dairy feeds	Zimbabwe	100
B3S3 b1	MZ569415	*Aspergillus niger* isolate *RUF-Asn*(MN944460)	Soil	Iran	100
B4S4 b	MZ569416	*Aspergillus fumigatus* strain *N16-1-5-1* (MN704716)	N/A	China	100
B3S2 b	MZ569417	*Aspergillus fumigatus* isolate *ZSY-5-4*(MT487775)	N/A	China	100
B3S2 A	MZ569418	*Aspergillus fumigatus* strain *N16-1-5-1* (MN704716)	N/A	China	100
B3S1 b	MZ569419	*Aspergillus terreus* strain *DTO 403-C9*(MT316343)	Compost pile	Italy	100
B3S3 b	MZ569420	*Aspergillus oryzae* strain *TF7*(MH625703)	Soil	Nigeria	100
B4S2	MZ569421	*Aspergillus fumigatus* strain *N15-2-1-1*(MN704714)	N/A	China	100
B3S4 b2	MZ569422	*Aspergillus fumigatus* strain *16FB* (MT267796)	Soil	Iran	100
B3S1 A	MZ569423	*Aspergillus fumigatus* isolate *RSO_SP17* (MN634626)	Drinking water	South Africa	100
B4S4	MZ569424	*Aspergillus nidulans* strain *DTO 402-H2*(MT316339)	Compost pile	Italy	100

**Table 5:** Enzyme activity of bacterial isolates.

**Isolate code**	**Accession no.**	**Enzymatic activities (diameter measured in mm)**				
		**Amylase**	**Cellulase**	**Esterase**	**Lipase**	**Gelatinase**
B2S2 b2	MZ571882	10.00 ± 1.15^e^	25.67 ± 1.20^c^	32.00 ± 1.16^b^	23.33 ± 1.76^f^	13.67 ± 0.88^d^
B3S4 A1	MZ571885	17.67 ± 0.33^c^	12.67 ± 1.45^b^	0.00 ± 0.00^a^	0.00 ± 0.00^a^	0.00 ± 0.00^a^
B2S1 A2	MZ571887	25.00 ± 5.13^g^	27.33 ± 0.67^c^	18.67 ± 0.67^d^	0.00 ± 0.00^a^	0.00 ± 0.00^a^
B2S4 A	MZ571899	0.00 ± 0.00^a^	0.00 ± 0.00^a^	72.67 ± 1.76^f^	14.33 ± 1.20^c^	22.00 ± 2.52^b^
B3S2 b	MZ571900	17.67 ± 0.88^c^	0.00 ± 0.00^a^	31.67 ± 2.03^b^	20.33 ± 0.88^bd^	34.67 ± 2.91^c^
B3S3 1	MZ571902	16.67 ± 2.40^c^	12.67 ± 1.45^b^	28.33 ± 2.03^b^	0.00 ± 0.00^a^	21.33 ± 1.33^b^
B3S3 2	MZ571903	9.00 ± 0.58^e^	21.00 ± 1.00^d^	0.00 ± 0.00^a^	0.00 ± 0.00^a^	0.00 ± 0.00^a^
B3S2a	MZ571905	20.00 ± 1.15^f^	12.67 ± 1.45^b^	0.00 ± 0.00^a^	0.00 ± 0.00^a^	13.00 ± 1.15^d^
B1S3 A	MZ571906	23.67 ± 0.88^fg^	13.33 ± 1.67^b^	0.00 ± 0.00^a^	0.00 ± 0.00^a^	0.00 ± 0.00^a^
B4S4 A1	MZ571911	7.33 ± 1.33^e^	0.00 ± 0.00^a^	0.00 ± 0.00^a^	0.00 ± 0.00^a^	12.00 ± 1.00^d^
B3S4 A2	MZ571881	0.00 ± 0.00^a^	46.67 ± 5.24^e^	0.00 ± 0.00^a^	0.00 ± 0.00^a^	0.00 ± 0.00^a^
B4S4 B1	MZ571883	11.67 ± 0.88^de^	37.00 ± 1.53^f^	53.00 ± 1.15^c^	0.00 ± 0.00^a^	22.00 ± 1.15^b^
B2S2 b	MZ571888	0.00 ± 0.00^a^	69.00 ± 2.65^g^	0.00 ± 0.00^a^	0.00 ± 0.00^a^	0.00 ± 0.00^a^
B3S4 b	MZ571913	0.00 ± 0.00^a^	0.00 ± 0.00^a^	12.00 ± 1.73^de^	18.00 ± 1.73^b^	21.00 ± 1.7^b^
B3S4A2	MZ571914	0.00 ± 0.00^a^	0.00 ± 0.00^a^	15.33 ± 1.76^d^	0.00 ± 0.00^a^	0.00 ± 0.00^a^
B3S1	MZ571907	13.00 ± 1.15^b^	28.00 ± 4.16^c^	16.33 ± 0.88^d^	0.00 ± 0.00^a^	0.00 ± 0.00^a^

*Note:* Mean values (n = 3) ± SEM. Values appended by different superscript letters within a row and column are significantly different (*p* < 0.05).

**Table 6:** Enzyme activity in fungal isolates.

**Isolate code**	**Isolate accession number**	**Amylase**	**Cellulase**	**Lipase**	**Esterase**	**Gelatinase**
B3S4 b	MZ569412	++	+++	−	−	++
B2S2 a1	MZ569413	++	++	−	−	++
B3S2 a	MZ569414	+++	+++	−	−	++
B3S3 b1	MZ569415	−	+++	−	−	+
B4S4 b	MZ569416	+++	++	−	−	+++
B3S1 b	MZ569419	++	++	−	−	−
B3S3 b	MZ569420	+++	+	−	−	++
B3S4 b2	MZ569422	++	+++	−	−	−
B3S1 A	MZ569423	++	++	−	−	+
B4S4	MZ569424	++	−	−	−	++

*Note:* Key: Enzyme activity in fungal isolates: (+++) high activity, (++) moderate activity, (+) low activity, and (−) no activity.

## Data Availability

The data that support the findings of this study are openly available in NCBI at https://ncbi.nlm.nih.gov/.

## References

[1] Abdel-Shafy H. I., Mansour M. S. (2018). Solid waste issue: sources, composition, disposal, recycling, and valorization. *Egyptian Journal of Petroleum*.

[2] Abubakar I. R., Maniruzzaman K. M., Dano U. L. (2022). Environmental sustainability impacts of solid waste management practices in the global south. *International Journal of Environmental Research and Public Health*.

[3] Massoud M. A., Mokbel M., Alawieh S., Yassin N. (2019). Towards improved governance for sustainable solid waste management in Lebanon: centralised vs decentralised approaches. *Waste Management & Research*.

[4] Anand S. (2010). *Solid waste management*.

[5] Demirbas A. (2011). Waste management, waste resource facilities and waste conversion processes. *Energy Conversion and Management*.

[6] Lissah S. Y., Ayanore M. A., Krugu J. K., Aberese-Ako M., Ruiter R. A. (2021). Managing urban solid waste in Ghana: perspectives and experiences of municipal waste company managers and supervisors in an urban municipality. *PLoS One*.

[7] Sharma R., Sharma M., Sharma R., Sharma V. (2013). The impact of incinerators on human health and environment. *Reviews on Environmental Health*.

[8] Webb H. K., Arnott J., Crawford R. J., Ivanova E. P. (2013). Plastic degradation and its environmental implications with special reference to poly (ethylene terephthalate). *Polymers*.

[9] Bardají D. K. R., Moretto J. A. S., Furlan J. P. R., Stehling E. G. (2020). A mini-review: current advances in polyethylene biodegradation. *World Journal of Microbiology and Biotechnology*.

[10] Singh G. K., Gupta K., Chaudhary S. (2014). Solid waste management: its sources, collection, transportation and recycling. *International Journal of Environmental Science and Development*.

[11] Han Z., Ma H., Shi G., He L., Wei L., Shi Q. (2016). A review of groundwater contamination near municipal solid waste landfill sites in China. *Science of the Total Environment*.

[12] Mukherjee S., Mukhopadhyay S., Hashim M. A., Sen Gupta B. (2015). Contemporary environmental issues of landfill leachate: assessment and remedies. *Critical Reviews in Environmental Science and Technology*.

[13] Siddiqua A., Hahladakis J. N., Al-Attiya W. A. K. (2022). An overview of the environmental pollution and health effects associated with waste landfilling and open dumping. *Environmental Science and Pollution Research*.

[14] Vaverková M. D. (2019). Landfill impacts on the Environment—Review. *Geosciences*.

[15] Sinha R. K., Valani D., Sinha S., Singh S., Herat S. (2009). Bioremediation of contaminated sites: a low-cost nature’s biotechnology for environmental clean up by versatile microbes, plants & earthworms. *Solid waste management and environmental remediation*.

[16] Saravanan A., Kumar P. S., Vo D.-V. N., Jeevanantham S., Karishma S., Yaashikaa P. (2021). A review on catalytic-enzyme degradation of toxic environmental pollutants: microbial enzymes. *Journal of Hazardous Materials*.

[17] Alemu F. (2015). Isolation and screening of protease enzyme producing bacteria from cheese at Dilla University, Ethiopia. *International Journal of Nutrition and Food Sciences*.

[18] Saxena S. (2015). Microbial enzymes and their industrial applications. *Applied microbiology*.

[19] Sharma B., Dangi A. K., Shukla P. (2018). Contemporary enzyme based technologies for bioremediation: a review. *Journal of Environmental Management*.

[20] Karigar C. S., Rao S. S. (2011). Role of microbial enzymes in the bioremediation of pollutants: a review. *Enzyme Research*.

[21] Bhandari S., Poudel D. K., Marahatha R. (2021). Microbial enzymes used in bioremediation. *Journal of Chemistry*.

[22] Gurung N., Ray S., Bose S., Rai V. (2013). A broader view: microbial enzymes and their relevance in industries, medicine, and beyond. *BioMed Research International*.

[23] Emmanuel U., Ifeanyichukwu I., Chika E., Chike O., Chinyere N. (2017). Isolation and characterization of bacteria and fungi associated with biodegradation of municipal solid wastes in Abakaliki Metropolis, Nigeria. *International Journal of Environment, Agriculture and Biotechnology*.

[24] Muhonja C. N., Makonde H., Magoma G., Imbuga M. (2018). Biodegradability of polyethylene by bacteria and fungi from Dandora dumpsite Nairobi-Kenya. *PLoS One*.

[25] Jain A., Yadav S., Nigam V. K., Sharma S. R. (2017). Fungal-mediated solid waste management: a review. *Mycoremediation and Environmental Sustainability*.

[26] Gautam S., Bundela P., Pandey A., Jamaluddin A. M. K., Sarsaiya S. (2012). Diversity of cellulolytic microbes and the biodegradation of municipal solid waste by a potential strain. *International Journal of Microbiology*.

[27] Lokhande S., Musaddiq M. (2014). Microflora degrading the municipal wastes by fungi. *Indian Journal of Life Sciences*.

[28] Partanen P., Hultman J., Paulin L., Auvinen P., Romantschuk M. (2010). Bacterial diversity at different stages of the composting process. *BMC Microbiology*.

[29] Shivlata L., Satyanarayana T. (2015). Thermophilic and alkaliphilic *Actinobacteria*: biology and potential applications. *Frontiers in Microbiology*.

[30] Zhao Y., Lu Q., Wei Y. (2016). Effect of actinobacteria agent inoculation methods on cellulose degradation during composting based on redundancy analysis. *Bioresource Technology*.

[31] Pramila R., Ramesh K. V. (2011). Biodegradation of low density polyethylene (LDPE) by fungi isolated from marine water a SEM analysis. *African Journal of Microbiology Research*.

[32] Radhakrishnan S., Varadharajan M., Dharumadurai D. (2022). Isolation, identification, and screening of polyene antifungal compound producing *streptomyces sampsonii* MDCE7 from Agroforestry Soil. *Methods in actinobacteriology*.

[33] Ismail Y., Yulvizar C., Mazhitov B. (2018). Characterization of lactic acid bacteria from local cow´s milk kefir. *IOP Conference Series: Earth and Environmental Science*.

[34] Tripathi N., Sapra A. (2020). *Gram staining*.

[35] MacWilliams M. P. (2012). *Indole test protocol*.

[36] Reiner K. (2010). Catalase test protocol. *American Society for Microbiology*.

[37] MacWilliams M. P. (2009). *Citrate test protocol*.

[38] McDevitt S. (2009). Methyl red and voges-proskauer test protocols. *American Society for Microbiology*.

[39] Nakei M. D. (2015). *Isolation and identification of plastics-degrading microorganisms From soils of Morogoro, Tanzania*.

[40] Peng Q., Wang X., Shang M. (2014). Isolation of a novel alkaline-stable lipase from a metagenomic library and its specific application for milkfat flavor production. *Microbial Cell Factories*.

[41] Lee L. P., Karbul H. M., Citartan M., Gopinath S. C., Lakshmipriya T., Tang T.-H. (2015). Lipase-secreting *Bacillus* species in an oil-contaminated habitat: promising strains to alleviate oil pollution. *BioMed Research International*.

[42] Alariya S. S., Sethi S., Gupta S., Lal G. B., Lal G. (2013). Amylase activity of a starch degrading bacteria isolated from soil. *Archives of Applied Science Research*.

[43] Shivani D., Kumar J. S. (2015). Extracellular enzymatic profile of fungal deteriogens of historical palace of Ujjain. *International Journal of Current Microbiology and Applied Sciences*.

[44] Dela Cruz T. E. E., Torres J. M. O. (2012). *Gelatin hydrolysis test protocol*.

[45] Sambrook J. (1989). *Molecular Cloning: A Laboratory Manual*.

[46] Huang L.-N., Zhou H., Zhu S., Qu L.-H. (2004). Phylogenetic diversity of bacteria in the leachate of a full-scale recirculating landfill. *FEMS Microbiology Ecology*.

[47] Saleemi M. K., Khan M. Z., Khan A. (2012). Molecular identification of black aspergilli isolated from poultry feeds by sequencing their ITS-regions. *Pakistan Veterinary Journal*.

[48] Ashelford K. E., Chuzhanova N. A., Fry J. C., Jones A. J., Weightman A. J. (2006). New screening software shows that most recent large 16S rRNA gene clone libraries contain chimeras. *Applied and Environmental Microbiology*.

[49] Altschul S. F., Gish W., Miller W., Myers E. W., Lipman D. J. (1990). Basic local alignment search tool. *Journal of Molecular Biology*.

[50] Saitou N., Nei M. (1987). The neighbor-joining method: a new method for reconstructing phylogenetic trees. *Molecular Biology and Evolution*.

[51] Kumar S., Stecher G., Li M., Knyaz C., Tamura K. (2018). MEGA X: molecular evolutionary genetics analysis across computing platforms. *Molecular Biology and Evolution*.

[52] Tamura K., Nei M., Kumar S. (2004). Prospects for inferring very large phylogenies by using the neighbor-joining method. *Proceedings of the National Academy of Sciences of the United States of America*.

[53] Felsenstein J. (1985). Confidence limits on phylogenies: an approach using the bootstrap. *Evolution*.

[54] Wang Q., Garrity G. M., Tiedje J. M., Cole J. R. (2007). Naive Bayesian classifier for rapid assignment of rRNA sequences into the new bacterial taxonomy. *Applied and Environmental Microbiology*.

[55] Mandal C., Tabassum T., Shuvo M. J., Habib A. (2019). Biochemical and molecular identification of antibiotic-producing bacteria from waste dumpsite soil. *Journal of Advanced Biotechnology and Experimental Therapeutics*.

[56] Danilova I., Sharipova M. (2020). The practical potential of bacilli and their enzymes for industrial production. *Frontiers in Microbiology*.

[57] Abubakar A., Abioye O., Aransiola S., Maddela N. R., Prasad R. (2024). Crude oil biodegradation potential of lipase produced by *Bacillus subtilis* and *Pseudomonas aeruginosa* isolated from hydrocarbon contaminated soil. *Environmental Chemistry and Ecotoxicology*.

[58] Prasad M., Manjunath K. (2011). *Comparative study on biodegradation of lipid-rich wastewater using lipase producing bacterial species*.

[59] Khandare S. D., Agrawal D., Mehru N., Chaudhary D. R. (2022). Marine bacterial based enzymatic degradation of low-density polyethylene (LDPE) plastic. *Journal of Environmental Chemical Engineering*.

[60] Asiandu A. P., Wahyudi A., Sari S. W. (2021). A review: plastics waste biodegradation using plastics-degrading bacteria. *Journal of Environmental Treatment Techniques*.

[61] Yamamoto-Tamura K., Hiradate S., Watanabe T. (2015). Contribution of soil esterase to biodegradation of aliphatic polyester agricultural mulch film in cultivated soils. *AMB Express*.

[62] Contesini F. J., Melo R. R., Sato H. H. (2018). An overview of *Bacillus* proteases: from production to application. *Critical Reviews in Biotechnology*.

[63] Chatterjee S., Sharma S., Prasad R. K. (2015). Cellulase enzyme based biodegradation of cellulosic materials: an overview. *Cellulose*.

[64] Akhtar N., Goyal D., Goyal A. (2015). Biodegradation of cellulose and agricultural waste material. *Advances in Biodegradation and Bioremediation of Industrial Waste*.

[65] Nanda S., Sahu S., Abraham J. (2010). Studies on the biodegradation of natural and synthetic polyethylene by *Pseudomonas* spp. *Journal of Applied Sciences and Environmental Management*.

[66] Savich V., Novik G. (2021). Waste biodegradation and utilization by Pseudomonas species. *Journal of Microbiology, Biotechnology and Food Sciences*.

[67] Sharma S., Pathak H. (2014). Pseudomonas in biodegradation. *International Journal of Pure & Applied Bioscience*.

[68] Lamilla C., Pavez M., Santos A., Hermosilla A., Llanquinao V., Barrientos L. (2017). Bioprospecting for extracellular enzymes from culturable Actinobacteria from the South Shetland Islands, Antarctica. *Polar Biology*.

[69] Al-Dhabi N. A., Esmail G. A., Ghilan A.-K. M., Arasu M. V. (2020). Isolation and screening of *Streptomyces* sp. Al-Dhabi-49 from the environment of Saudi Arabia with concomitant production of lipase and protease in submerged fermentation. *Saudi Journal of Biological Sciences*.

[70] Amore A., Pepe O., Ventorino V., Birolo L., Giangrande C., Faraco V. (2012). Cloning and recombinant expression of a cellulase from the cellulolytic strain Streptomyces sp. G12 isolated from compost. *Microbial Cell Factories*.

[71] Gohain A., Manpoong C., Saikia R., De Mandal S. (2020). Actinobacteria: diversity and biotechnological applications. *Recent advancements in microbial diversity*.

[72] Zada S., Xie J., Yang M. (2021). Composition and functional profiles of microbial communities in two geochemically and mineralogically different caves. *Applied Microbiology and Biotechnology*.

[73] Anastasi A., Varese G. C., Filipello Marchisio V. (2005). Isolation and identification of fungal communities in compost and vermicompost. *Mycologia*.

[74] Rouhullah D., Mohammad Ali A., Esmail C. (2012). Identification of fungal communities in producing compost by windrow method. *Journal of Environmental Protection*.

[75] Krikstaponis A., Lugauskas A., Krysinska-Traczyk E., Prazmo Z., Dutkiewicz J. (2001). Enzymatic activities of *Aspergillus fumigatus* strains isolated from the air at waste landfills. *Annals of Agricultural and Environmental Medicine*.

[76] El Barnossi A., Moussaid F., Iraqi H. (2019). Study of mycoflora associated with the decomposition of solid green household waste in the natural environment. *Asian Journal of Microbiology, Biotechnology and Environmental Sciences*.

[77] Cihangir N., Sarikaya E. (2004). Investigation of lipase production by a new isolate of *Aspergillus s*p. *World Journal of Microbiology and Biotechnology*.

[78] Contesini F. J., Lopes D. B., Macedo G. A., da Graça Nascimento M., de Oliveira Carvalho P. (2010). Aspergillus sp. lipase: potential biocatalyst for industrial use. *Journal of Molecular Catalysis B: Enzymatic*.

[79] Basheer S. M., Chellappan S., Beena P., Sukumaran R. K., Elyas K., Chandrasekaran M. (2011). Lipase from marine *Aspergillus awamori* BTMFW032: production, partial purification and application in oil effluent treatment. *New Biotechnology*.

[80] Sethi B. K., Nanda P. K., Sahoo S. (2016). Characterization of biotechnologically relevant extracellular lipase produced by *Aspergillus terreus* NCFT 4269.10. *Brazilian Journal of Microbiology*.

[81] Muhammad B. A. O. A. A., Okiki P. A. (2016). Cellulase production by fungi isolated from Odo-Aremu dumpsite in Ado-Ekiti, Nigeria. *Enzyme*.

[82] Ogbonna C., Okpokwu N., Okafor C., Onyia C. (2014). Isolation and screening of amylase producing fungi obtained from garri processing site. *International Journal of Biotechnology and Food Science*.

[83] Abdullah R., Shaheen N., Iqtedar M., Naz S., Iftikhar T. (2014). Optimization of cultural conditions for the production of alpha amylase by Aspergillus niger (BTM-26) in solid state fermentation. *Pakistan Journal of Botany*.

[84] Asrat B., Girma A. (2018). Isolation, production and characterization of amylase enzyme using the isolate Aspergillus niger FAB-211. *International Journal of Biotechnology and Molecular Biology Research*.

[85] Ratnasri P., Lakshmi B., Ambika Devi K., Hemalatha K. (2014). Isolation, characterization of Aspergillus fumigatus and optimization of cultural conditions for amylase production. *International Journal of Research in Engineering & Technology*.

[86] Su N., Lee M. (2001). Screening and characterization of koji molds producing saline-tolerant protease. *Journal of Industrial Microbiology and Biotechnology*.

[87] Liang Y., Pan L., Lin Y. (2009). Analysis of extracellular proteins of Aspergillus oryzae grown on soy sauce koji. *Bioscience, Biotechnology, and Biochemistry*.

[88] Oyeleke S., Egwim E. C., Auta S. (2010). Screening of Aspergillus flavus and Aspergillus fumigatus strains for extracellular protease enzyme production. *Journal of Microbiology and Antimicrobials*.

[89] Martiny A. C. (2019). High proportions of bacteria are culturable across major biomes. *The ISME Journal*.

[90] Atieno B. (2023). *Bioprospecting for potential useful microorganisms from Mwakirunge dumpsite in Mombasa County, Kenya*.

